# Role of Working Temperature and Humidity in Acetone Detection by SnO_2_ Covered ZnO Nanowire Network Based Sensors

**DOI:** 10.3390/nano12060935

**Published:** 2022-03-12

**Authors:** Fanny Morisot, Claudio Zuliani, Mireille Mouis, Joaquim Luque, Cindy Montemont, Tony Maindron, Céline Ternon

**Affiliations:** 1 Univ. Grenoble Alpes, CNRS, Grenoble INP (Institute of Engineering, Univ. Grenoble Alpes), LMGP, F-38000 Grenoble, France; morisotf@gmail.com; 2 Univ. Grenoble Alpes, CNRS, Grenoble INP (Institute of Engineering, Univ. Grenoble Alpes), IMEP-LAHC, F-38000 Grenoble, France; mireille.mouis@grenoble-inp.fr; 3 AMS Sensors UK Limited, Deanland House, Cowley Road, Cambridge CB4 0DL, UK; claudio.zuliani1977@gmail.com (C.Z.); jluquear@gmail.com (J.L.); 4 Univ. Grenoble-Alpes, CEA-LETI, MINATEC Campus, 17 Rue des Martyrs, CEDEX 9, F-38054 Grenoble, France; cindy.montemont@laposte.net (C.M.); tony.maindron@minalogic.com (T.M.)

**Keywords:** ZnO nanowire networks, SnO_2_ sensing layer, acetone detection, humidity effect, temperature effect, microhotplate

## Abstract

A randomly oriented nanowire network, also called nanonet (NN), is a nano-microstructure that is easily integrated into devices while retaining the advantages of using nanowires. This combination presents a highly developed surface, which is promising for sensing applications while drastically reducing integration costs compared to single nanowire integration. It now remains to demonstrate its effective sensing in real conditions, its selectivity and its real advantages. With this work, we studied the feasibility of gaseous acetone detection in breath by considering the effect of external parameters, such as humidity and temperature, on the device’s sensitivity. Here the devices were made of ZnO NNs covered by SnO_2_ and integrated on top of microhotplates for the fine and quick control of sensing temperature with low energy consumption. The prime result is that, after a maturation period of about 15 h, the devices are sensitive to acetone concentration as low as 2 ppm of acetone at 370 °C in an alternating dry and wet (50% of relative humidity) atmosphere, even after 90 h of experiments. While still away from breath humidity conditions, which is around 90% RH, the sensor response observed at 50% RH to 2 ppm of acetone shows promising results, especially since a temperature scan allows for ethanol’s distinguishment.

## 1. Introduction

The presence of acetone in breath is a relevant biomarker for the measurement of insulin-deficient catabolic states in diabetic patients [1,2]. Indeed, when the body consumes its lipid reserves due to a lack of sugar, it synthesizes ketone bodies, which is the case, for example, of diabetics in crisis. Acetone is expelled via respiration and is then found in non-negligible quantities (>20 ppm) in breath, whereas the usual quantities are around 400 ppb [3]. Exhaled breath has a complex composition that changes with activity, such as smoking or consuming alcohol. This has already been exploited with breath tests used to detect alcohol consumption. It is also influenced by physiology, for instance, in the case of hypoglycemia resulting from diet or diabetes. Thus, monitoring the composition of breath, or at least the presence of given elements, may allow the monitoring of a patient’s health. Apart from acetone (a biomarker for hypoglycemia, diabetic crisis and metabolic issues leading to obesity), and even without being exhaustive, human breath may still contain ethanol (a marker for alcohol consumption with the usual amount being in the range of 100–200 ppb, which reaches >5 ppm in the case of alcohol consumption) or nitric oxide (indicator for an inflammation of the respiratory tract, NO reacting very quickly with O_2_ to form NO_2_, which is the final biomarker, with usual amount around 20 ppb which reaches >0.1 ppm in the state of inflammation) [4,5]. In addition, the humidity level of human breath is in the range of 90–95% relative humidity (RH) [6].

Most gas sensors exploit the chemiresistive effects that occur in metal oxide materials (MOX) such as ZnO or SnO_2_ powders when they are in contact with a gas [7]. This chemiresistive effect results from redox reactions between the target gas and surface adsorbed oxygen ions, which induces a change in carrier concentration in the sensing material and, hence, a change in its conductivity or resistivity. The electrical response is thus governed by the process of oxidation and reduction at the metal oxide surface. Heating is required as this process is based on thermally activated reactions where penetration of gas molecules is dependent on the temperature of the sensing material. For breath detection of ketones, such as acetone, ZnO nanoparticles and other similar oxide-based materials are typically used [8,9,10,11,12].

The sensitivity of solid-state gas sensors strongly depends on their microstructure. It can be improved if the surface-to-volume ratio of the material used for the sensing is increased. With nanostructures of small diameters (less than twice the Debye length [10], which ranges from 10 to 100 nm for moderately doped oxides [13]), changes in surface charge density, induced for instance by redox reactions at the surface, are able to control carrier concentration in the whole volume of the nanostructure [9,14]. Therefore, alternative methods based on novel nanomaterials are emerging. For example, Chakraborathy et al. propose sonochemically prepared nanosized γ-Fe_2_O_3_ sensors for detecting sub-ppm of acetone against a background of simulated human breath [15]. Ferroelectric WO_3_ nanoparticles have also been shown to detect acetone with good selectivity in breath-simulated media [16]. Sensors consisting of SiO_2_ thin films doped with WO_3_ nanoparticles were able to selectively detect acetone concentrations as low as 20 ppb [17]. However, although they are based on nanoparticles, these materials are quite compact. Their response is relatively slow because the process involves gas infiltration at grain boundaries, which takes significant time. Being based on powders, they cannot be suspended either. In contrast, nanowires (NWs) are uniquely suitable for sensor applications because of their single-crystalline structure, large surface-to-volume ratio, and high stability. Metal oxide nanowire-based sensors have already been demonstrated for detecting gases. Particularly, a chemiresistor based on electrospun metal oxide nanowires has been able to detect acetone with good selectivity in breath-simulated media [18]. With nanonets, as proposed in this work, it is even possible to envision the fabrication of suspended networks over contact pads of adequate topology.

Usual metal oxide sensors require high operating temperatures in the range of about 150–400 °C [10], which adds significantly to power consumption when using a conventional alumina sensing platform. In order to overcome this, local microheaters based on microelectromechanical systems (MEMS) and several combinations of metal oxide particles with other nano-structures have been evaluated. Here, by using micro-hotplates, which were specially developed for local heating, power consumption was radically reduced. For instance, the heater can reliably reach temperatures of 400 °C (resp. 250 °C) with 50 mW (resp. 30 mW) of power in less than 15 ms and cool down to ambient temperature in about 30 ms [19,20].

Moreover, as described in the literature, a major problem in gas sensing remains selectivity as most metal oxides used for a specific target gas are also sensitive to some other volatile species. One proposed solution is based on crossing the breath response of several materials, each operating at its optimum temperature.

In a previous study [21], we have described the impact of aluminum-doped ZnO (AZO) encapsulation and temperature on acetone detection by ZnO NN on microheaters. In a dry atmosphere, the detection ability was as low as 1.5 ppm of acetone at 370 °C, but the lack of sensitivity to acetone in a humid atmosphere was a bottleneck challenge regarding the objective of breath analysis (RH ≈ 90%). In this study, in order to resolve the issue related to humidity, new encapsulation materials were studied. The studied sensors were composed of a composite material, the SnO_2_ layer present on the surface provides sensitivity to acetone, and the underlying nanowire network provides conduction and mobility. The expected results should have good sensitivity and rapid response combined with moderate energy consumption. Deposition of either doped or undoped 6 nm-thick SnO_2_ thin films was achieved on top of the ZnO NNs. The performance of the sensors based on these encapsulated NNs was studied in comparison with that of sensors based on bare ZnO NNs. This study is a first, but important, step towards the detection of acetone in breath, with the aim to validate the experimental elements one by one and thus lead to the most relevant experimental protocol for a future complete study of the acetone biosensor, which is beyond the scope of this paper. We varied sensor material characteristics (NN density and encapsulation layer) and carefully explored the role of three parameters on the detection: (1) atmosphere humidity (0% RH and 50% RH), (2) microhotplate temperature (230 °C, 300 °C and 370 °C) and (3) response to different gases (acetone, ethanol, dioxide nitride). A possible explanation of the observed phenomena is given in relation to the laws explained in the section on the gas sensing mechanisms.

## 2. Gas Sensing Mechanisms

In the absence of extrinsic doping, the conductivity of metal oxides (MOX) is controlled by oxygen vacancies in bulk (n-type conduction) and by charged species adsorbed on the surface and, more specifically, $O_{2}^{-}$, $O^{-}$, $O^{2-}$. According to the literature review conducted by Barsan, and as demonstrated by various techniques (infrared analysis, temperature-programmed desorption and electron paramagnetic resonance), the presence of these species is temperature-dependent [22]. Although some authors question the mechanisms of formation of these different species [23], recent literature still claims their presence and, in particular, Yamozoe et al. [24] have recently studied the kinetics of formation of these surface charged species. Thus, we present here a synthesis of the latest literature data in terms of temperature, kinetics, and impact of humidity on the latter, on which we will base the interpretation of our experimental results. When the temperature is below 150 °C, the dominant oxygen species on the surface of SnO_2_ are $O_{2}^{-}$ according to the reaction (1); between 150 °C and 320 °C, the $O^{-}$ species are dominant, according to the reaction (2) whose kinetic is high [24]. Above 320 °C, $O^{-}$ and $O^{2-}$ species coexist, according to reactions (2) and (3) respectively. The kinetics of reaction (3) is very low [25].

(1) $$T\;<\;150{\;}^{{}^{\circ}}C\;\;O_{2}+e^{-}⇌O_{2}^{-}\;\;K1,\;$$

(2) $$T\;>\;150{\;}^{{}^{\circ}}C\;\;O_{2}+2e^{-}⇌2O^{-}\;\;\;K2,\;\mathrm{high}\;\mathrm{kinetics}$$

(3) $$T\;>\;320{\;}^{{}^{\circ}}C\;\;O_{2}+4e^{-}⇌2O^{2-}\;\;\;K3,\;\mathrm{low}\;\mathrm{kinetics}$$

Thus, depending on the temperature, the adsorption of oxygen on the surface according to the reactions (1), (2) and (3) is accompanied by the capture of one to four electrons from the volume of the semiconductor. So, in the presence of oxygen, the resistance of SnO_2_ increases under the effect of the charged species at the surface. Furthermore, tin oxide is semiconducting, hence an increase in temperature results in a sharp increase in the intrinsic carrier concentration. Thus, for high temperatures, an undoped tin oxide can feature the same conductivity as a low-doped tin oxide when the intrinsic carrier concentration reaches the dopant one level. As a conclusion, in the frame of gas sensing, the conductivity of the active material is influenced by temperature, by the presence of charged species on the surface and by their density.

Furthermore, MOX materials, including tin oxide and ZnO, are very sensitive to the presence of moisture in the air, as water reduces the surface charged oxygens to form hydroxyl groups, a phenomenon called “hydroxyl poisoning” [24,25]. According to Grossmann et al. [26], the only reaction to occur in the presence of water (4) is accompanied by the release of electrons into the material, which implies a decrease in resistance.

(4) $$H_{2}O+{\;O}^{-}\;\left({\mathrm{or}\;O}^{2-}\right)+2\mathrm{Sn}\;⇌2\mathrm{SnOH}+{\;e}^{-}\;\left(\mathrm{or}\;2e^{-}\right)\;K4$$

This is, in fact, the same phenomenon that underlies the gas detection capability of MOX. On the one hand, an oxidizing gas, such as NO_2_, will have the same effect as oxygen by increasing the negatively charged species on the surface, which implies an increase in the resistance. On the other hand, a reducing gas, such as ethanol or acetone, will have the same effect as water by reducing the charged oxygen species on the surface, which results in a decrease in resistance.

It is then obvious that the presence of oxygen or water in the atmosphere to be analyzed will have a significant impact on detection. Our ultimate research perspective is to detect acetone in breath, a medium rich in oxygen and water. It is therefore important to take proper account of the effect of these two elements on the material and its electrical behavior.

Many studies have been carried out on this issue, and by compiling the literature [24,25,26,27], it appears that water acts in three different manners on SnO_2_ and its detection ability. Firstly, the presence of water completely inhibits the (3) reaction, which implies that only $O^{-}$ species are present on the surface [24,25,27]. Secondly, a recent study [25] shows that temperature ageing in a humid atmosphere leads to a “maturing” of the surface. As described in Table 1, maturation is characterized by the fact that the equilibrium constants K2_wet/dry_ and K3_wet/dry_ change when the material is exposed to water for a long time at a high temperature. K2_dry_, K3_dry_ and K2_wet_ increase, whereas K3_wet_ decreases, further inhibiting the (3) reaction. Subsequently, the equilibrium of reaction (2) is shifted to the right irrespective of the atmosphere, whereas the equilibrium of reaction (3) is shifted to the right only in a dry atmosphere, the (3) reaction being totally inhibited in a humid atmosphere [25]. As a consequence, the density of charged species on the metal oxide surface increases in comparison with non-maturated surfaces. Moreover, the new surface density achieved is maintained even after returning to a dry atmosphere [25]. Table 1 summarizes the impact of this ageing on the equilibrium constants of reactions (2) and (3) at 350 °C when the ageing is carried out at 580 °C, either in a dry atmosphere or under P_H_2_O_ = 0.05 atm. Therefore, when ageing is carried out in a humid atmosphere, the sensitivity of the material to reducing gases increases due to a higher density of $O^{-}\;$(and $O^{2-}$) on the surface and their capability of reacting with the reducing gas.

In this study, we are interested in two reducing gases, acetone and ethanol, and an oxidizing gas, NO_2_. The main mechanisms proposed for acetone [28] and ethanol [29] involve their degradation to form CO_2_ and water (reactions (5) and (6)), whereas for NO_2_ [25], as for oxygen, it is simple adsorption (reaction (7)).

(5) $${\mathrm{CH}}_{3}-\mathrm{CO}-{\mathrm{CH}}_{3}+8O^{-}\;\left(8O^{2-}\right)⇌3{\mathrm{CO}}_{2}+3H_{2}O+8e^{-}\;\left(16e^{-}\right)\;K5$$

(6) $$\mathrm{Et}-\mathrm{OH}+6O^{-}\;\left(6O^{2-}\right)⇌2{\mathrm{CO}}_{2}+3H_{2}O+6e^{-}\;\left(12e^{-}\right)\;\;\;\;\;K6$$

(7) $${\mathrm{NO}}_{2}+e^{-}⇌{\mathrm{NO}}_{2}^{-}\;\;\;\;\;\;\;\;\;\;\;\;\;\;\;\;\;\;\;K7$$

Due to the formation of water as a product of reactions (5) and (6), H_2_O can thus react according to reaction (4) and induce hydroxyl poisoning of the surface.

In our study, the sensor was maintained at a given temperature, under a gas flow alternating exposure to the gas under study (in the presence of water or not) and exposure to the reference atmosphere (humid or dry air). The parameter of interest that was measured to evaluate the detection was the resistance of the sensor -*R_a_* under air and *R_g_* under gas-, which varies with the exposure as described above. The measured resistance was then used to calculate the sensor response (*S*), which was defined as the ratio between the sensor electrical resistance under air (*R*_a_) to under gas (*R*_g_) for reducing gases or the ratio between *R*_g_ and *R*_a_ for oxidizing gases (Table 2). The gas was considered detected if sensor response (the ratio) was larger than 1.

While sensor response has been initially determined empirically [30], studies have been carried out in recent years to relate it to sensor response on the basis of semiconductor physics, reaction mechanisms and measurement conditions [22,24,31]. In general, for a low concentration of the gas to be detected (<100 ppm), it appears that sensor response can be expressed in the simplified form [30]:

(8) $$S=\frac{R_{a}}{R_{g}}=1+k\left(T\right){\left[gas\right]}^{b}$$

with *b*, the power law exponent and *k*(*T*), a prefactor called sensitivity coefficient, thermally activated according to an Arrhenius’ law (*E_a_* activation energy, *k*_0_ prefactor, *k_B_* Boltzman’s constant, *T* temperature) [30]:

(9) $$k\left(T\right)=k_{0}\times exp\left(-\frac{E_{a}}{k_{B}T}\right)$$

## 3. Materials and Methods

### 3.1. Device Fabrication

**Figure 1:**
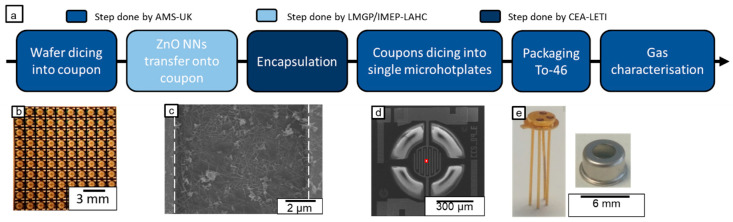
(**a**) Scheme presenting the main steps of device fabrication and packaging as done by each partner, (**b**) Optical image of a coupon, (**c**) SEM image of an encapsulated NN, (**d**) SEM image of a microhotplate and (**e**) Optical image of To-46 packaging.

**Figure A1:**
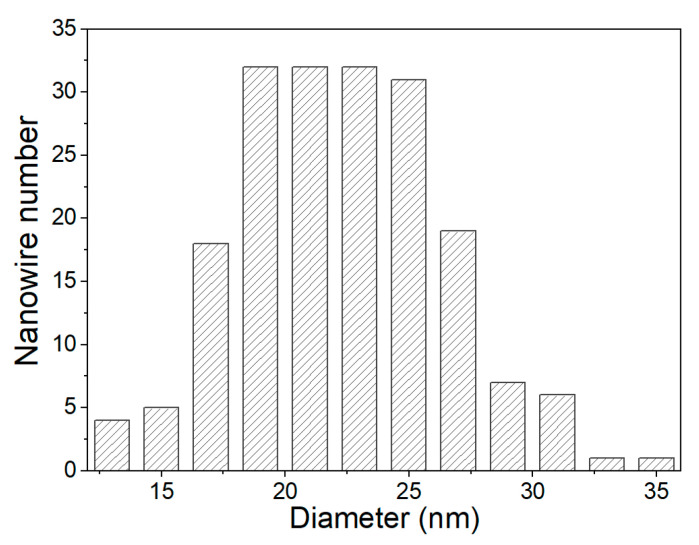
Statistics relative to the diameter of ZnO NWs in nanonet. The total of nanowire measured is 188.

The entire process of fabrication has already been detailed in previous articles [21,32,33]. It is the result of a close collaboration between two academic labs (LMGP/IMEP-LAHC), an RTO (CEA-LETI) and an industrial company (AMS-UK). The main steps of the NN integration on top of the microheaters with an interdigitated electrode array (IDA) are described in Figure 1, along with the partner involved (Figure 1a). The inter-electrode distance was fixed at 10 μm. The total surface of the microhotplate was 0.13 mm^2^ (200 μm in diameter). First, 200 mm industrial wafers of microhotplates were diced in pieces of 1 cm^2^ called coupons, which contained around 100 microhotplates (Figure 1b,d). In parallel, monocrystalline ZnO NWs, with a mean diameter of 22 nm (Figure A1 in Appendix A) and mean length of 1.5 µm, were assembled in a randomly oriented nanowire network, also called nanonets (NN), by filtration on a nitrocellulose membrane. These nanonets consist of networks of randomly oriented nanowires. They were transferred on top of the coupons by the dissolution of the membrane in acetone (Figure 1c). The morphological parameters of the NWs have been studied previously by X-ray diffraction (XRD), scanning electron microscopy (SEM) and high resolution transmission electron micrscopy (HRTEM) as described in [21,33]. Here, in order to enhance NN stability, an optional encapsulation layer was deposited after NN transfer by atomic layer deposition. As described in previous work [32], SnO_2_ was deposited at 150 °C with a chamber pressure of 0.2 Torr from Tetrakis(dimethylamino) tin (IV) (TDMASn) and H_2_O_2_ or H_2_O (0.1 s pulse) for doped and undoped SnO_2_ respectively (Table 3). To finish the integration process, each microhotplate was singularized and bonded in a To46 package before performing gas characterizations, which are developed in the following section. An image of the To46 packaging is shown in Figure 1e.

**Figure A2:**
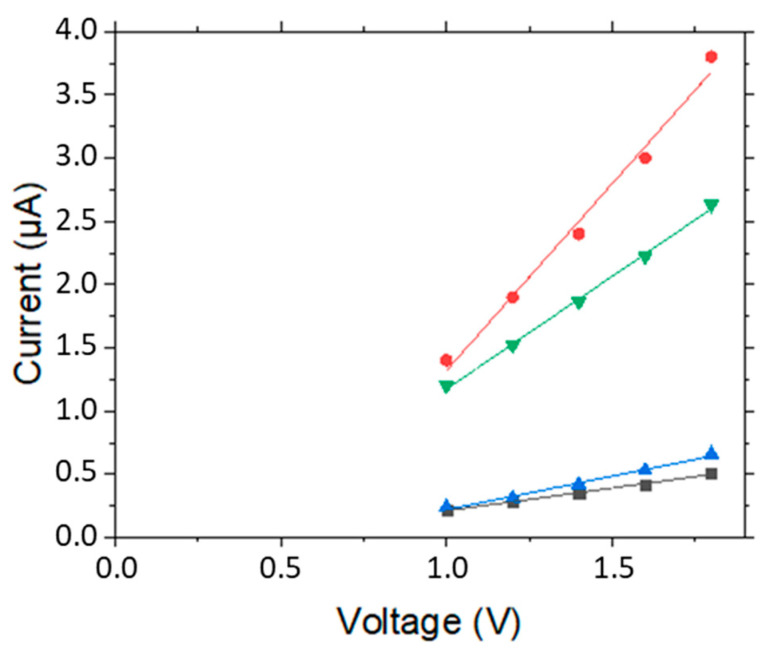
I–V curves of four bare devices tested at 370 °C.

Table 3 summarizes all the different samples that are presented in this work. On the one hand, in order to directly evaluate encapsulating layer’s effects, reference devices without encapsulation, called bare devices, were fabricated and tested. On the other hand, in order to explore the impact of the SnO_2_ encapsulation conductivity on detection, two different materials were used for NN based sensor encapsulation. Namely, D-type devices were encapsulated with doped SnO_2_ (doped), while I-type ones were encapsulated with undoped tin oxide (intrinsic). For the purpose of reproducibility investigation, four devices of each type were included in each study. Before any gas testing, the linearity of current-voltage (I–V) characteristics was first confirmed. Typical results are shown in Appendix B (Figure A2).

### 3.2. Gas Exposure

**Figure 2:**
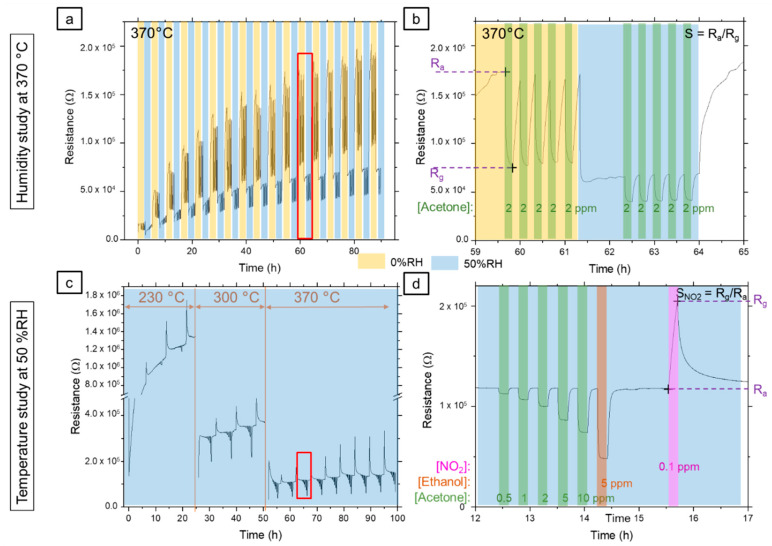
Typical evolution of one D_Low_ device’s electrical resistance for the whole gas exposure sequence (**a**,**c**) and for one cycle of exposure while presenting the method to calculate sensor response (**b**,**d**) used in this work for “Humidity Study” (**a**,**b**) and “Temperature Study” (**c**,**d**). As described in Table 2, sensor response is the ratio between electrical resistances when under air (*R_a_*) and when under gas (*R_g_*) for acetone and ethanol exposure. For dioxide nitride exposure, sensor response is the ratio between *R_g_* and *R_a_*.

Gas exposure was done using in-house built equipment at AMS-UK. In a first step, called “Humidity Study”, humidity impact on the sensors was evaluated by exposing them to 2 ppm of acetone, alternatively in dry (0 % RH) and humid (50 % RH) atmosphere at 370 °C. The test sequence used for this run is described in Table 4, gas exposure details are compiled in Table 5, and one typical “Humidity Study” run is illustrated in Figure 2a. As the detection stability of our device is an important parameter, we chose to repeat the cycle 17 times, leading to an experiment duration of about 90 h. Moreover, each cycle was made of five successive injections of 2 ppm acetone in a dry atmosphere and eventually five successive injections of 2 ppm acetone in a humid atmosphere, each injection separated by an air purge as described in Table 4 and Table 5. In a second step, called “Temperature Study”, the effects of microhotplate temperature and the exposure to different gases were studied on a new sample set. This study was done in a 50% RH humidity atmosphere as described in Table 4, whereas the gas exposure sequence is reported in Table 5. Three sensing temperatures were chosen in the linear working regime of the microheaters: 230 °C, 300 °C and 370 °C. For each temperature, we studied the effect of detection temperature on the three chosen gases: ethanol, acetone and dioxide nitride (NO_2_). In addition, five concentrations of acetone ranging from 0.5 to 10 ppm were studied (Table 5). Again, between each gas injection, an air purge was realized, and for each temperature, several cycles were conducted. It is to be noted that between each temperature cycle, devices were cooled down to ambient temperature. Typical electrical resistance evolution of one device during one “Temperature study” run is plotted in Figure 2c,d.

## 4. Results and Discussion

Each sensor described in the previous section was fully investigated according to the two different experimental protocols called “Humidity Study” and “Temperature Study” also described above and whose typical overview for one sensor response is shown in Figure 2. However, for the purpose of clarity and as long as the same behavior is observed for other devices, each time of relevance, solely D_Low_-type device performances will be discussed here.

### 4.1. Device Aging

In Figure 2, by considering the global electrical behavior of one device along the 90 h of the experiment duration, three main phenomena were observed.

Firstly, as observed clearly in Figure 2a for the “Humidity Study”, the resistance baseline was increasing drastically in the early stage of the experiment (from the first wet cycle until about 30 h) before stabilization. On the basis of the work of Suematsu et al. [25] and as described in Section 2, such behavior can be explained by the effect of ageing at high temperatures in the presence of water. We did not cure the devices voluntarily, however, due to the experimental conditions, at each wet cycle, each device spent 150 min at 370 °C in the presence of water (50% RH). Although the temperature was lower than that presented in ref [25] for maturation, it can be reasonably assumed that similar phenomena occurred, probably with slower kinetics which could explain the evolution of the resistance over the first 30 h of the experiment. Then, at a high temperature, the presence of water resulted in an increase in the density of negatively charged surface species ($O^{-}$ and $O^{2-}$ depending on the atmosphere) until saturation. With such an increased density, in the presence of oxygen in dry air, more electrons were captured on the surface by the oxygen species, leading to a depletion layer and an increase in the resistance.

Secondly, as expected, the resistance was larger in dry than in the wet atmosphere. Indeed, as a reducing agent, H_2_O reacts with surface oxygen species according to the (4) reaction producing electrons going back to the material, with a simultaneous decrease in the depletion layer in the semiconductor (These two phenomena are detailed and schematically illustrated in Section 4).

Thirdly, from the “Temperature Study”, Figure 2c, one can observe that resistance decreased when device temperature increased. This phenomenon is due to the well-known thermal activation of semiconducting materials. Upon temperature increase, due to thermal agitation, more and more carriers are able to reach the conduction band from the valence band. This leads to an increase in the intrinsic carrier concentration, which can exceed that of doping induced extrinsic carriers, decreasing material resistivity. The material behaves then as if it was intrinsic.

In the following sub-sections, we will first focus on the “Humidity Study” cycles at 370 °C in order to study the stability of the sensor response and to determine the role played by the encapsulation layer on this response. By comparing the dry and humid areas of the cycle, the role played by humidity is also explored. Thereafter, we will explore the impact of the temperature on sensor response, sensitivity coefficient and selectivity.

### 4.2. Sensor Response Stability

Sensor response stability refers to the device’s ability to produce the same results under the same exposure conditions. On the one hand, this can be tested by making several successive exposures without changing the external conditions such as the microhotplate’s temperature or the humidity in the exposure chamber. This is the case within the dry or humid sub-cycles, where sensors are exposed to five successive acetone pulses. On the other hand, this can be tested by doing successive exposures under the same conditions separated by a change in external conditions. This is the case from one cycle to the next; between two dry sub-cycles, a humid sub-cycle is intercalated and reciprocally.

**Figure 3:**
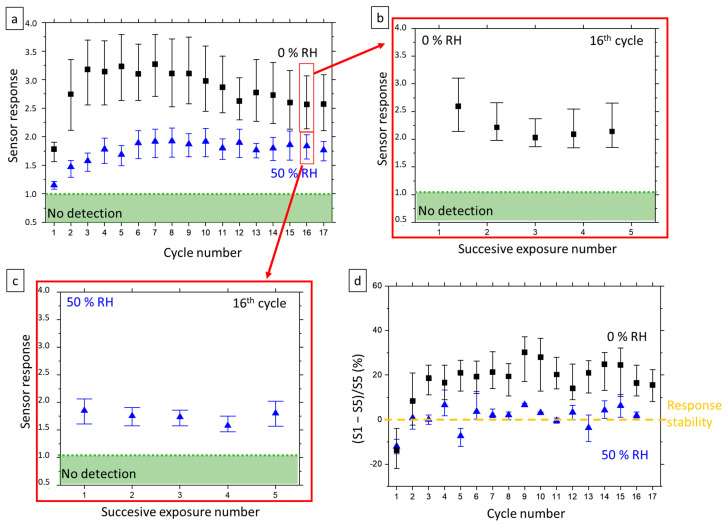
Evolution of D_Low_ devices response in “Humidity Study” (**a**) for the first exposure to 2ppm of acetone under dry and 50% RH humid atmosphere for the whole study, (**b**) during the 16th exposure cycle under dry atmosphere and (**c**) during the 16th exposure cycle under 50% RH. (**d**) Relative loss of sensor response between the first (S1) and the fifth (S5) successive injection of acetone for all 17 gas exposure cycles in “Humidity Study”. Black squares refer to results in dry atmosphere and blue triangle in humid atmosphere. The symbol represents the mean response calculated from 4 devices and the upper and lower lines represent respectfully the value of higher and lower responses obtained. Dashed line in panel (**d**) refers to stability of the response towards successive injections of acetone. Red rectangles in Figure 2a represent the 16th cycle which is detailed in Figure 2b,c. Data for I_Low_ loss of response are presented in Appendix C (Figure A3).

**Figure 4:**
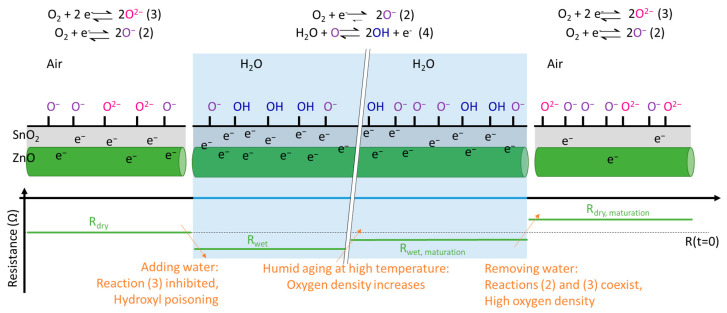
Schematic representation of phenomenon occurring during the experimental sequence, taking account of the water-oxygen interplay as described in Section 2. Detailed explanations are given in the text Section 4.3. This diagram is an adaption from Suematsu et al. [25].

Figure 3a shows the evolution of sensor response for the D_Low_-type devices, calculated from the first exposure to 2 ppm of acetone at 370 °C during the 17 cycles of the whole gas exposure sequence (=90 h). Dark square data points were calculated under dry conditions and blue triangle ones under 50% RH humidity. As expected, due to the hydroxyl poisoning (reaction (4)), and as explained in Section 2, the presence of water in the atmosphere implies a sharp decrease in device response between dry and wet sub-cycles. In the first half of the run, both dry and wet responses behave similarly, with a significant increase in sensor response over the first cycles of exposure (≈20 h), followed by stabilization. In the second half of the run (from ≈50 h), the wet response is stable until the end of the experiment, while the dry response slowly decreases. As explained in Section 2, such an increase in sensor response at the beginning of the run is directly linked to the wet ageing [25]. Due to the combined effect of temperature and humidity, even though intermittently applied, the density of charged species on the metal oxide surface increases and offers more reduction sites for acetone, thus leading then to an improved sensor response (See Figure 4 for a schematic representation of these phenomena).

The second observation in Figure 3a is that dispersion in sensor response is less important when devices are measured under a humid atmosphere. One hypothesis for this behavior is based on the presence of different surface oxygen species. Because of modified kinetics, in the case of the wet atmosphere, only $O^{-}\;$ (reaction (2)) can be formed on the surface, while in the case of the dry atmosphere, $O^{-}\;$ and $O^{2-}$ (reactions (2) and (3)) coexist. Then, upon acetone exposure, the number of electrons released under the effect of surface reduction depends on which species is reduced, thus inducing intrinsic experimental variability in the case of the dry atmosphere. In a humid atmosphere, a single species is present on the material surface, thus offering less dispersion of the results, as observed experimentally.

In dry measurement conditions, the observed behavior is rather good in terms of stability. Indeed, the slow degradation of the response ability occurs for a very long time with gas and humidity exposure (>50 h of continuous cycling). Such long-term stability had also been demonstrated in the literature for sensors based on SnO_2_ nanowires [34]. By contrast, for humid measurement conditions, after an initial increase due to the humid ageing of SnO_2_ surface, the observed behavior is excellent in terms of stability, which is very encouraging with regard to the objective of detecting acetone in breath since the breath has a moisture content of 90% RH. Then, in relation to its intended use, i.e., a few minutes of exposure per measurement carried out by a patient as part of the monitoring of acetone, the sensor lifetime would be more than 6 years.

Figure 3b,c reports the sensor response values for the five acetone injections during the dry and the wet parts of the 16th cycle of exposure (one cycle = 150 min), respectively. Figure 3d exposes the loss of sensor response between the first injection (S1) and the fifth injection (S5) ((S1 − S5)/S5) for wet and dry atmospheres over the whole experiment. Thus, for measurements in a dry atmosphere, there is a loss in sensor response of about 20% between the 1st and 5th injection, while in a humid atmosphere, the stability of response is observed. Moreover, these behaviors are confirmed all along the 90 h of measurements. This loss in sensor response is undoubtedly originating from the kinetics of the reactions in dry conditions. Indeed, as can be observed in Figure 2b, in the dry part of the cycle, 15 min are not sufficient to reach the steady state, particularly for recovering, whereas the same time is sufficient for humid conditions. Recovery involves both reactions (2) and (3) to produce $O^{-}\;$ and $O^{2-}$ surface species, and as described in the literature [24], (3) is a very slow reaction. As a consequence, the recovery of the sensor is not fully achieved upon the following injection, and fewer oxygen-surface species are available for reduction by acetone, leading to a decrease in sensor response.

As a consequence, in the following result analysis, only the first exposure of each cycle is considered for sensor response calculation, and the first cycles, dedicated to sensor maturation, are no more taken into account for future explanations.

### 4.3. Water-Oxygen Interplay at Tin Oxide Surface

Understanding the role played by water vapor and oxygen on the detection mechanism is fundamental to deciding on the architecture of the sensors and optimizing their operation. Based on the literature review described in Section 2 and the experimental results from Section 4.1 and Section 4.2, we are able to propose a model of the interaction between water, oxygen tin oxide surface, electron concentration and their impact on the resistance of the sensor. This model is schematically shown in Figure 4.

Before surface maturation, in dry air, both (2) and (3) reactions occur in parallel, (2) being dominant. As a result, the SnO_2_ surface contains $O^{-}$ as the dominant species and $O^{2-}$ in a lower density that capture electrons from the core of the material, resulting in a given resistance R_dry_. By the addition of water, reaction (3) is inhibited, and hydroxyl poisoning occurs (reaction (4)), releasing electrons in the material. As a consequence, the resistance of the material (R_wet_) decreases and a low density of $O^{-}$ is present at the surface. Then, because of humid ageing at a high temperature and as described above (Table 1), the equilibrium of (2) reaction is shifted to the right, leading to a persistent increase in $O^{-}$ surface species density, implying an increase in the material resistance (R_wet,maturation_). Finally, when exposed again to dry air, SnO_2_ surface is back to $O^{-}$ and $O^{2-}$ as surface species, but with increased density, implying an increased resistance (R_dry, maturation_ > R_dry_).

**Figure 5:**
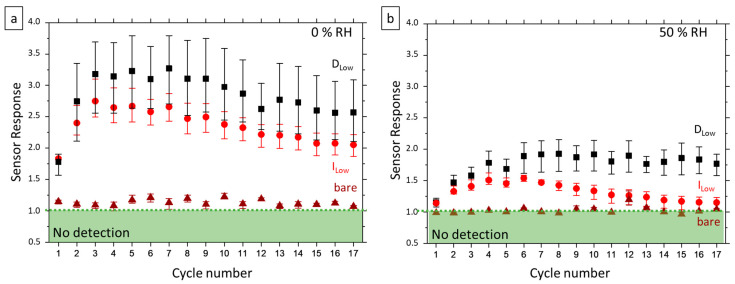
Evolution of D_Low_ (black square), I_Low_ (red circle) and bare (brown triangle) devices response for the whole “Humidity Study” (**a**) under dry atmosphere and (**b**) under 50% RH humid atmosphere. The sensor response is determined from the first exposure to 2 ppm of acetone. The symbol represents the mean calculated from 4 D_Low_ devices and 3 I_Low_ and bare devices. The upper and lower lines represent respectfully the higher and lower sensor responses calculated.

As a consequence, with an increased density of surface charged species, the reaction sites for gas detection are more numerous, and sensitivity is increased after surface maturation. Because of hydroxyl poisoning in the wet atmosphere, reaction sites for gas detection are reduced, and sensitivity in wet conditions is always smaller than in dry air. Finally, due to the concomitant existence of two charged species on the surface, there appears to be greater variability in the sensitivity in a dry atmosphere, as each act of acetone detection can release 8 or 16 electrons into the material (reaction (5)). By contrast, in the wet atmosphere, the same number of electrons is always released, and sensitivity is highly reproducible, as observed in Figure 3 and Figure 5.

### 4.4. Effect of Encapsulating Layer

Figure 5 presents the evolution of sensor response to the first injection in the dry (Figure 5a) and wet (Figure 5b) part of each exposure cycle over the whole gas sequence for three types of devices with different encapsulation but the same nanowire density of 5 × 10^8^ NW cm^−2^. Bare refers to devices without encapsulation, while D_Low_ and I_Low_ stand for doped and undoped SnO_2_ encapsulating layer, respectfully.

Prime observation is that the response of bare devices is quite low, near the limit of detection, for dry measurement and null for wet measurement, while encapsulated devices show a good response towards 2 ppm of acetone whatever the atmosphere (dry and wet) and for both types of encapsulation. This behavior can be explained by two phenomena. Firstly, the amount of sensitive surface is totally different in encapsulated and non-encapsulated sensors. We defined the amount of sensitive surface as the ratio between the full developed sensing surface and the footprint of the active region, between the electrodes, covered by the sensing material. For a perfectly flat surface, the amount of sensitive surface equals 1. In the presence of texturing, the amount of sensitive surface exceeds 1, while for nanonets, the amount of sensitive surface can be smaller than 1 when only NWs do contribute to sensing. Here, for non-encapsulated sensors, the amount of sensitive surface is that of nanowires alone (3.11 × 10^−1^ for NWs 22 nm in diameter and 1.5 µm in length, with a density of 5 × 10^8^ NW cm^−2^) whereas in the case of encapsulated sensors, the entire surface of SnO_2_, increased by the texturing due to nanowires, which can thus contribute to sensing (so that the amount of sensitive surface reaches 1.11, with same NW parameters). Moreover, although both materials are metal oxides, they can respond differently to the presence of acetone on the surface, SnO_2_ being quite widely used for gas sensing applications such as ethanol [35] or acetone [36]. Between encapsulated devices, it is to note that D-type devices exhibit higher sensitivity than I-type ones. This difference can come from additional oxygen vacancies present in the D-type encapsulating layer with its intentional doping compared to I-type encapsulating layer (Table 3) [37]. As a consequence, with more charges, the D encapsulating layer can react more and thus, the device’s sensitivity is higher. For both types of encapsulation, sensitivity is lower in humid conditions; the three first cycles correspond to surface maturation and the sensors’ sensitivity decreases slowly for a long time in the dry atmosphere, as observed previously. However, solely the D encapsulating layer leads to stable sensitivity in the wet atmosphere.

### 4.5. Sensor Characterization

In this part, based on the previous results, we study the response of the sensors to various concentrations of acetone at different working temperatures in a humid atmosphere (50% RH) (“Temperature Study”, see Figure 2c,d for a general overview). Cross sensitivity to ethanol and nitrogen dioxide is also explored. So, sensors based on low-density nanonets with D-type and I-type SnO_2_ encapsulation layer (D_Low_ and I_Low_) are submitted to the second gas exposure sequence, which is detailed in Table 5 for 3 different temperatures as detailed in Table 4. These temperatures were chosen in the linear working range of the microheater: 230 °C, 300 °C and 370 °C. According to the previous part, the first cycles were dedicated to sensor maturation; thus, sensor response was calculated from the first injection of the third cycle of exposure at 230 °C and 300 °C and the sixth one at 370 °C.

#### 4.5.1. Acetone Sensitivity

**Figure 6:**
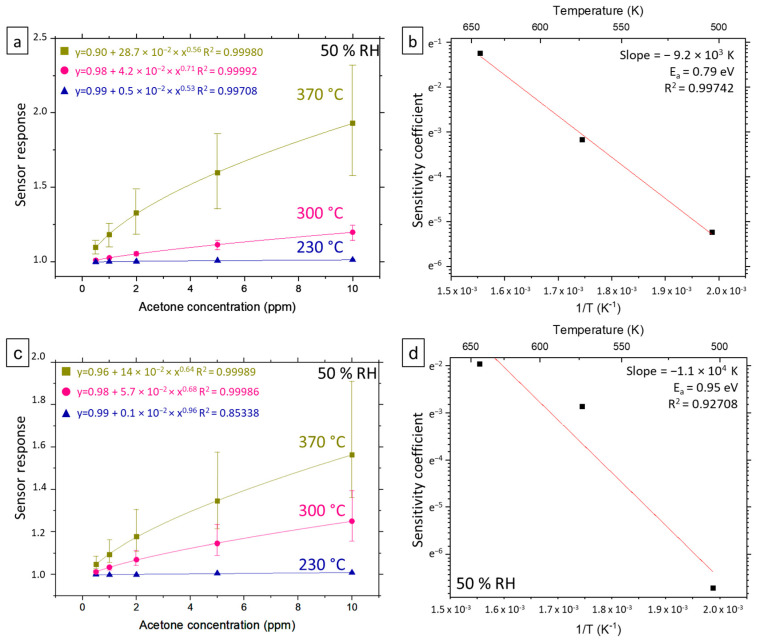
(**a**) (resp. (**c**)) Evolution of D_Low_ (resp. I_Low_) devices response towards acetone under 50% RH humid atmosphere and different working temperature. The symbol represent the mean calculated from 4 (resp. 3) devices and the upper and lower lines represent respectfully the higher and lower sensor response calculated. Sensor response values were calculated from the third cycle of exposure at 230 °C and 300 °C and the sixth one at 370 °C. Allometric fits were done in accordance with Equation (8). (**b**,**d**) Evolution of sensitivity coefficient *k*(*T*) as a function of inverse temperature.

Figure 6a exhibits the response of D_Low_ sensors as a function of acetone concentration in the range of 0.5–2 ppm for the three microheater temperatures. The first observation is that the response to acetone increases with temperature, which is coherent with the thermal activation of our semiconductor-based device. At 230 °C, the sensor is no longer able to detect lower acetone concentrations (<2 ppm), while concentrations as low as 0.5 ppm are detected from 300 °C. Each response curve was fitted in accordance with Equation (8), with good results. The sensitivity coefficients, *k*(*T*), for acetone at each of the operating temperatures and as deduced from the fitting parameters are displayed in Figure 6b. The graph in log-scale shows the gas sensitivity coefficient against inverse temperature. The straight line results from a linear fit of the data and indicates an exponential dependence on inverse temperature, as expected from Equation (9). As a consequence, the deduced activation energy of the sensitivity coefficient for acetone is 0.79 eV. As shown in Figure 6c,d, the same behavior and the same order of magnitude for energy activation (0.95 eV) are observed for the I_Low_ sensors. No value for the same type of sensors is available in the literature. However, the activation energy for acetone sensing is found for different materials in the same order of magnitude. As an example, Kao and coworkers [38] inferred an activation energy of 0.95 eV from a study dealing with acetone detection from an indium nitride (InN) gas sensor.

#### 4.5.2. Sensor Selectivity

**Figure 7:**
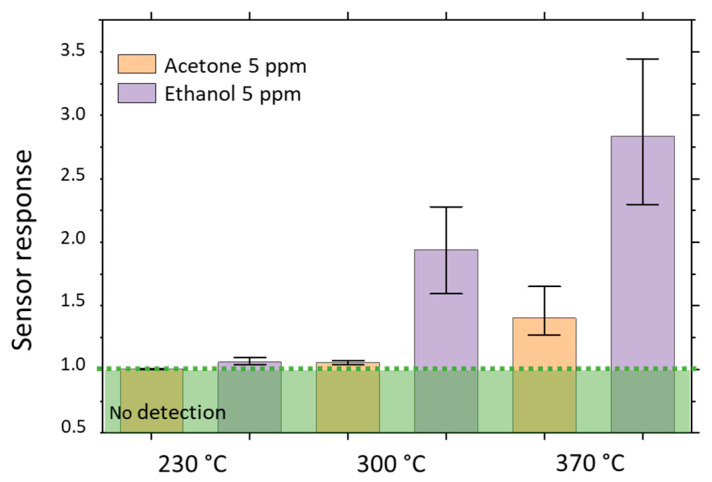
D_Low_ devices response calculated at 230 °C, 300 °C and 370 °C to 5 ppm of acetone and 5 ppm of ethanol under 50% RH humid atmosphere. The top of the columns represents the mean response calculated from 4 devices and the upper and lower lines represent respectively the higher and lower value of sensor response obtained. Sensor response values were calculated from the third cycle of exposure at 230 °C and 300 °C and the sixth one at 370 °C.

**Figure A4:**
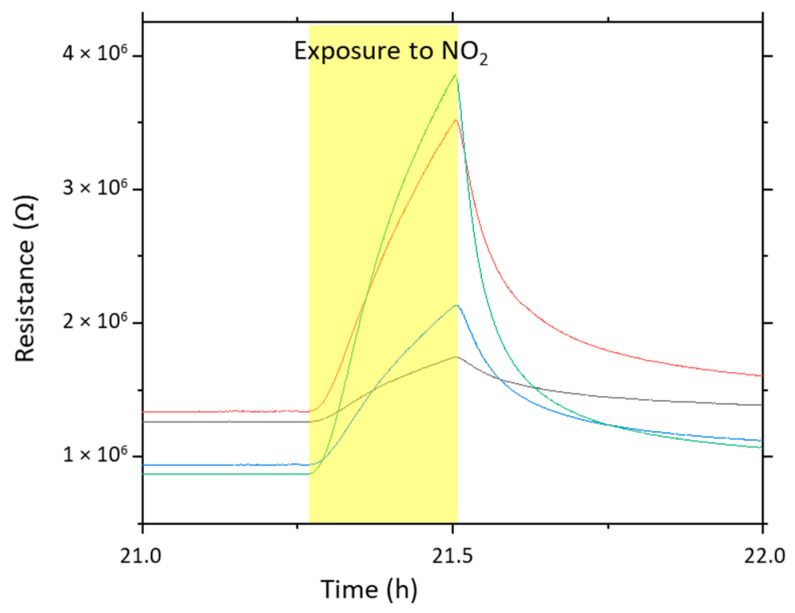
Typical electrical resistance evolution of DLow devices when exposed to dioxide nitride (0.1 ppm) at 370 °C.

As selectivity is an important parameter for a gas sensor, in this part, we explore the D_Low_ device’s ability to detect other gases than acetone. As the application objective of these sensors is the detection of acetone in the breath, we have chosen to study two other gases that may also be present in the breath, as described in the gas sequence (Table 4): 5 ppm of ethanol and 0.1 ppm of dioxide nitride which are respectively the signature for alcohol absorption and inflammation of the respiratory tract [4,5]. Moreover, ethanol, similar to acetone, is a reducing gas, whereas NO_2_ is an oxidizing one. As shown in Figure 2c,d, our sensors are highly sensitive to NO_2_, but as the response time to this gas is too long hence the device’s electrical resistance was not able to stabilize itself within the 15 min of exposure (Figure 1d and Figure A4 in Appendix D). This phenomenon of not reaching stabilized electrical resistance under NO_2_ is quite known for n-type MOX gas sensors at high temperatures and has already been reported in the literature [39,40]. As a consequence, sensor response calculation does not make sense as a steady state is not reached and solely sensor response towards ethanol compared to the same concentration of acetone as a function of detection temperature is presented in Figure 7.

Response toward acetone and ethanol is higher if the detection temperature is set to 370 °C, as previously observed. However, for a given temperature, the response towards ethanol is higher than towards acetone, suggesting lower activation energy for ethanol. As usual for MOX based gas sensors, if we rely on a single measurement, our devices are not selective toward acetone [41,42]. However, thanks to the different activation energies, it is possible to discriminate between ethanol and acetone by playing on the working temperature, which is easy with the microhotplates as the basis of the sensors. On the one hand, in a sequential mode, the first measurement at 230 °C would show whether ethanol is present inside the tested mix of gas. Then, if no ethanol is present, the measurement at 370 °C gives the information concerning acetone. On the other hand, in a parallel mode, two similar sensors are simultaneously sensing the gas; one is driven at 230 °C and the other at 370 °C. Here again, the sensing of acetone is meaningful only if the sensor driven at 230 °C detects no ethanol. Hence, sequential (on one sensor) or parallel (at the same time on two sensors) measurements could be implemented to be sure of acetone detection. An example of parallel gas detection is presented on SnO_2_ thin film by Sysoev and et al. [43]. The use of microhotplates allowing local heating at different temperatures is an asset for potential integration in a sensing array.

Then, sequential (on one sensor) or parallel (at the same time on two sensors) measurements could be implemented to be sure of acetone detection. Typically, a measurement at 230 °C enables the presence or absence of ethanol to be determined; then, if no ethanol is present, the measurement at 370 °C gives the information concerning acetone.

## 5. Conclusions

In this work, we presented results concerning the detection of acetone in different humidity conditions (dry or 50% RH) for devices based on ZnO nanonets coated with SnO_2_. The first part of this work allowed us to demonstrate the excellent stability of the developed devices, at least 90 h at 370 °C under variable humid atmosphere. Surprisingly, the best stability and the lowest dispersion of the measurements was obtained when the atmosphere was humid, which is an advantage when the object of analysis is breath.

By studying the morphology and composition of the sensor, we have shown that in the range studied that the encapsulation layer is essential for acetone detection in a humid atmosphere. Moreover, with a low-doping level, the SnO_2_ encapsulation layer allows a better sensitivity and stability is obtained. The experimental results from the Humidity Study were found to be consistent with works previously published in the literature. The observed trends could be explained by the interplay of water vapor and oxygen and their impact on sensor response evolution, its stability and its variability. After ageing at a high temperature in the presence of water, the sensitivity of the sensor was improved whatever the atmosphere. Then, when the measurement was done in a wet atmosphere, the stability was enhanced, and variability was reduced. The second part of this work focused on the role of the operating temperature and its impact on the detection of acetone in variable concentrations, as well as ethanol and nitride dioxide. Whatever the gas was, an increase in temperature resulted in an increase in sensitivity. Furthermore, we have shown that the evolution of sensor response with the concentration of acetone correctly followed an allometric-type law, which has enabled us to deduce the activation energy for the detection of acetone. Although our devices were more sensitive to ethanol than acetone, this study showed that discrimination between the two gases is possible by combining two measurements, parallel or successive, at two different detection temperatures.

Thus, the results obtained with gas sensors based on ZnO nanonets coated with low-doped tin oxide are extremely promising. Indeed, they offer the possibility to detect acetone at concentrations as low as 0.5 ppm in a humid atmosphere (50% RH). Moreover, discrimination between acetone and ethanol is possible by playing on the sensor temperature. In addition, thanks to the developed architecture, based on micro-hotplates, the working temperature, in the range 230–370 °C, is quickly reached with moderate energy consumption while allowing a rapid change from one temperature to another.

## Figures and Tables

**Table 1:** Summary of equilibrium constants of oxygen adsorption under dry and wet atmosphere at 350 °C and the effect of ageing under dry and wet atmosphere at 580 °C on those parameters. Values of those parameters have been extracted from Suematsu et al. [25].

(2) and (3) Parameters		Measurement @ 350 °C in Dry Air		Measurement @ 350 °C in Wet Air (P_H_2_O_ = 0.012 atm)		For a Given Ageing Atmosphere, Impact of Water on Surface Species:
		K2_dry_	K3_dry_	K2_wet_	K3_wet_	
Aging @ 580 °C	Dry Atmosphere During Aging	8 × 10^−11^	8 × 10^−41^	1 × 10^−11^	3 × 10^−49^	In Presence of Wet Atmosphere: K2 ↘: Density of O^−^ ↘, K3 ↘↘: O^2−^ blocked
	Wet Atmosphere During Aging	5 × 10^−7^	3 × 10^−36^	4 × 10^−11^	7 × 10^−50^	
**For A Given Measurement Atmosphere, Impact of Water in the Ageing Atmosphere on Surface Species:**		When aging is done in wet atmosphere: *K2_wet/dry_ and K3_dry_↗: equilibria are shifted to the right*				
		$K2\;↗↗:\;\mathrm{Density}\;\mathrm{of}\;O^{-}\;$ ↗↗ $K3\;↗↗:\;\mathrm{Density}\;\mathrm{of}\;O^{2-}\;$↗↗ $\mathrm{With}\;d(O^{2-}$$)\;<<\;d(O^{-}$)		$K2\;↗:\;\mathrm{Density}\;\mathrm{of}\;O^{-}\;$ ↗, $K3\;↘:\;O^{2-}$ more blocked		

**Table 2:** Sensor response definition, as used in this work, and defined as a function of the nature of the gas to be detected. Gas is detected if sensor response (the ratio) is higher than 1. *R_a_*, resistance under air and *R_g_*, under gas.

	Reducing Gas Acetone/Ethanol	Oxidizing Gas NO_2_
Sensor Response (*S*)	$\frac{R_{a}}{R_{g}}$	$\frac{R_{g}}{R_{a}}$

**Table 3:** Summary of all the different type of devices based on ZnO NN investigated in this work.

	Bare	D_Low_	I_Low_
Encapsulation by ALD	none	doped SnO_2_	undoped SnO_2_
Oxidizing Agent	-	H_2_O_2_	H_2_O
Reducing Agent	-	TDMA(VI)Sn	TDMA(VI)Sn
Layer Thickness (nm)	-	6	6
Layer Conductivity (Ω^−1^ cm^−1^) at Room Temperature	-	0.1	0
Nanowire Density (NW cm^−2^)	5 × 10^8^	5 × 10^8^	5 × 10^8^
Number of Functional Devices/Tested Devices (Humidity Study)	4/4	4/4	3/4
Number of Functional Devices/Tested Devices (Temperature Study)	NA	4/4	4/4

**Table 4:** Test sequence parameters for Humidity and Temperature Studies. For clarity purpose, the gas sequences (nature, concentration, number of injections, exposure time and purge time) are detailed in Table 5.

	Humidity Study		Temperature Study		
Name	Dry/Humid		230	300	370
Number of cycles	17		3	3	9
Sub-cycle	Dry	Humid	-	-	-
Humidity (% RH)	0	50	50		
Temperature (°C)	370		230	300	370
Number of Gas Injection in One Cycle (Each Injection is Separated by a Purge)	5	5	7		
Stabilization Time in Cycles Before 1st Acetone Injection (min)	60		60		

**Table 5:** Summary of gas injections and their respective concentration, number of injections, exposure and purge time used in humidity and temperature studies.

	Humidity Study		Temperature Study		
Sub Cycle	Dry	Humid	For Each Temperature		
Gas Sequence	Acetone	Acetone	Acetone	Ethanol	NO_2_
Concentration (ppm)	2	2	0.5/1/2/5/10	5	0.1
Number of Injection (Each One is Separated by a Purge)	5	5	5 (one at each concentration)	1	1
Exposure Time/Purge Time (min)	15/15	15/15	15/15	15/30	15/30

**Figure A3:**
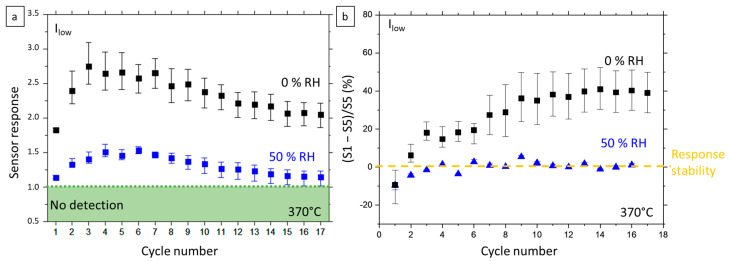
Evolution of DLow devices response in “Humidity Study” (**a**) for the first exposure to 2 ppm of acetone under dry and 50% RH humid atmosphere for the whole study, (**b**) Relative loss of sensor response between the first (S1) and the fifth (S5) successive injection of acetone for all 17 gas exposure cycles in “Humidity Study”. Black squares refer to results in dry atmosphere and blue triangle in humid atmosphere. The symbol represents the mean response calculated from 3 devices and the upper and lower lines represent respectfully the value of higher and lower responses obtained. Dashed line in panel b refers to stability of the response towards successive injections of acetone.

## Data Availability

The data presented in this study are available on request from the corresponding author.

## Appendix A. Statistics Relative to the Diameter of ZnO NWs in Nanonet

Figure A1 the statistics relative to the nanowire diameter leading to the mean diameter 22 nm and standard deviation 4 nm.

## Appendix B. Sensor Response Linearity

Figure A2 presents typical I–V curves of studied devices. It clearly shows that even though there is a disparity of electrical resistance between devices, each curve is linear.

## Appendix C. Sensor Response Stability

Figure A3 presents the evolution of the decrease in sensor response between the first injection (S1) and the fifth injection (S5) of one sub-cycle ((S1 − S5)/S5) for wet and dry atmospheres over the whole experiment for I_Low_ devices during “Humidity Study” (black squares under dry atmosphere and blue triangle under 50% RH humidity). We retrieve the same observations as for D_Low_ devices: sensor response stabilization under wet atmosphere and loss of sensitivity under dry atmosphere. The decrease in sensor response was more important for I_Low_ devices, (around 40% after 17 cycles of exposure) than for D_Low_ devices (around 20%).

## Appendix D. Sensor Selectivity

Figure A4 shows the evolution of electrical resistance of D_Low_ devices when exposed to NO_2_. It is clearly observed that, even after 15 min of exposure, devices resistance has not stabilized. Thus, it cannot be compared to the response towards acetone and ethanol, which was calculated based on stabilized values of the electrical resistance under gas exposure.
